# Gold aggregating gold: A novel nanoparticle biosensor approach for the direct quantification of hepatitis C virus RNA in clinical samples

**DOI:** 10.1016/j.bios.2016.11.001

**Published:** 2017-06-15

**Authors:** Sherif M. Shawky, Ahmed M. Awad, Walaa Allam, Mohamed H. Alkordi, Sherif F. EL-Khamisy

**Affiliations:** a Center of Genomics, Helmy institute, Zewail City of Science and Technology, Sheikh Zayed Dist., 12588 Giza, Egypt; b Krebs Institute, Department of Molecular Biology and Biotechnology, University of Sheffield, Sheffield S10 2TN, UK; c Misr University for Science and Technology, Faculty of Pharmacy, Biochemistry Department, Giza, Egypt; d Center for Materials Science, Zewail City of Science and Technology, Sheikh Zayed Dist., 12588 Giza, Egypt

**Keywords:** HCV, Nucleic acid, Gold nanoparticles, RNA detection, DNA repair, Topoisomerase

## Abstract

The affordable and reliable detection of Hepatitis C Virus (HCV) RNA is a cornerstone in the management and control of infection, affecting approximately 3% of the global population. However, the existing technologies are expensive, labor intensive and time consuming, posing significant limitations to their wide-scale exploitation, particularly in economically deprived populations. Here, we utilized the unique optical and physicochemical properties of gold nanoparticles (AuNPs) to develop a novel assay platform shown to be rapid and robust in sensing and quantifying unamplified HCV RNA in clinical samples. The assay is based on inducing aggregation of citrate AuNPs decorated with a specific nucleic acid probe. Two types of cationic AuNPs, cysteamine and CTAB capped, were compared to achieve maximum assay performance. The technology is simple, rapid, cost effective and quantitative with 93.3% sensitivity, high specificity and detection limit of 4.57 IU/µl. Finally, our data suggest that RNA folding impact the aggregation behavior of the functionalized AuNPs, with broader applications in other nucleic acid detection technologies.

## Highlights

• Direct detection of unamplified HCV RNA in clinical samples.
• The Biosensor platform is dependent on the RNA folding behavior.
• The developed platform is qualitative, quantitative, and independent on the pH and/or the source of the sample.
• The biosensor platform could be easily automated.
• First time to use gold nanoparticles in inducing aggregation of gold nanoparticles for unamplified nucleic acids detection and quantification.

## Graphical abstract

**Figure:**
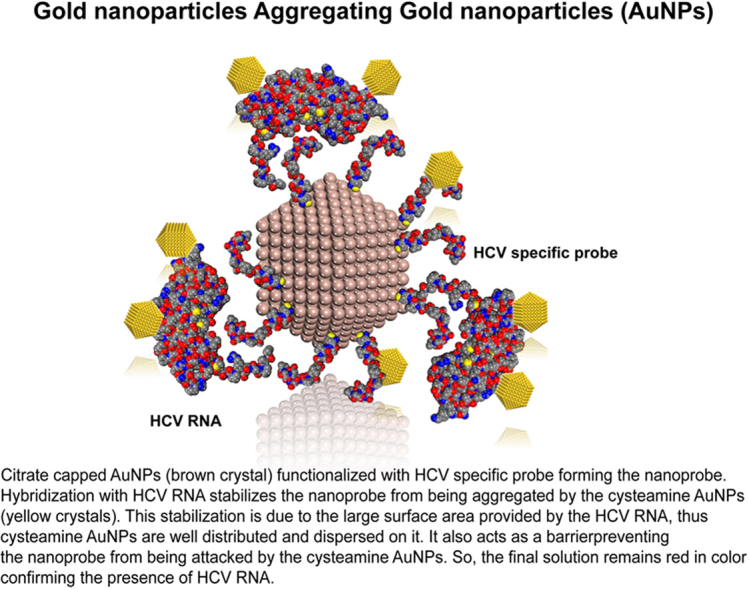

## 1 Introduction

Approximately 3% of the world population are infected with hepatitis C virus (HCV) with 3–4 million infections annually and at least 150 million chronic carriers at risk of developing liver cirrhosis and/or liver cancer (WHO, 2016). It was estimated that more than 15% of the Egyptian population are currently infected with HCV (Hajarizadeh et al., 2013; Mauss et al., 2013) with almost half million new cases arising annually. The infection usually progresses to fatty liver and hepatocellular carcinoma, posing significant health and economic challenges to the society (Miller and Abu-Raddad, 2010). Since an approved vaccination against HCV is yet to be established (Lee et al., 2015, WHO, 2016), the prime combating strategies rely on newly developed medications coupled to robust and affordable means of viral detection and quantification (AASLD-IDSA, 2016, Seifert et al., 2015).

HCV diagnosis is achieved by serologic and Nucleic Acid Testings (NATs) (Scott and Gretch, 2007). A major drawback of the serological approach is the inability to detect acute infections (Cloherty et al., 2016) and the associated complications of immuno-suppression patients (Atrah and Ahmed, 1996; Mauss et al., 2013). NATs are based mainly on Real-Time RT-PCR, branched- DNA (b-DNA), and transcription-mediated Amplification (TMA). NATs are relatively expensive, labor intensive, and require adequately equipped labs, posing significant limitations to their point of care testing. Thus, alternative approaches for HCV RNA detection and quantification are urgently needed.

The unique optical properties of gold nanoparticles (AuNPs), were originated from their strong Surface Plasmon Resonance (SPR) phenomena, which is responsible for their intense colors, and high extinction coefficient compared to conventional dyes (Huang et al., 2007a). Thus, AuNPs have been employed in many colorimetric assays for different biological molecules as proteins (Kim et al., 2009), and nucleic acids (Liandris et al., 2009). For example, Mirkin and co-workers were the first to develop a modified AuNPs cross-linking method for the direct detection of nucleic acids (Elghanian et al., 1997, Larguinho et al., 2015). Despite the high sensitivity and specificity of this method, it requires firm temperature control for precise target detection. To our knowledge, this method has not been used for nucleic acids detection clinically. Li and co-workers developed a method for the direct detection of nucleic acids using unmodified AuNPs (Li and Rothberg, 2004a, Li and Rothberg, 2005). The technique is based on the adsorption behavior of single and double stranded nucleic acid onto AuNPs surface and the medium ionic strength. Despite its sensitivity and specificity, it requires precise control of the probe, salt, and AuNPs concentrations.

The non-cross linking method was first introduced by Sato and co-workers (Sato et al., 2003), which is based on functionalizing AuNPs with single stranded thiol-modified probe. AuNPs aggregation is based also on the ionic strength of the medium. However, the desired results are only achieved if the target is of the same length as the probe. Baptista and co-workers improved this technique to detect long nucleic acids; however it still depends on either high ionic strength or the pH to induce AuNPs aggregation (Baptista et al., 2008, Larguinho et al., 2015).

**Fig. 1:**
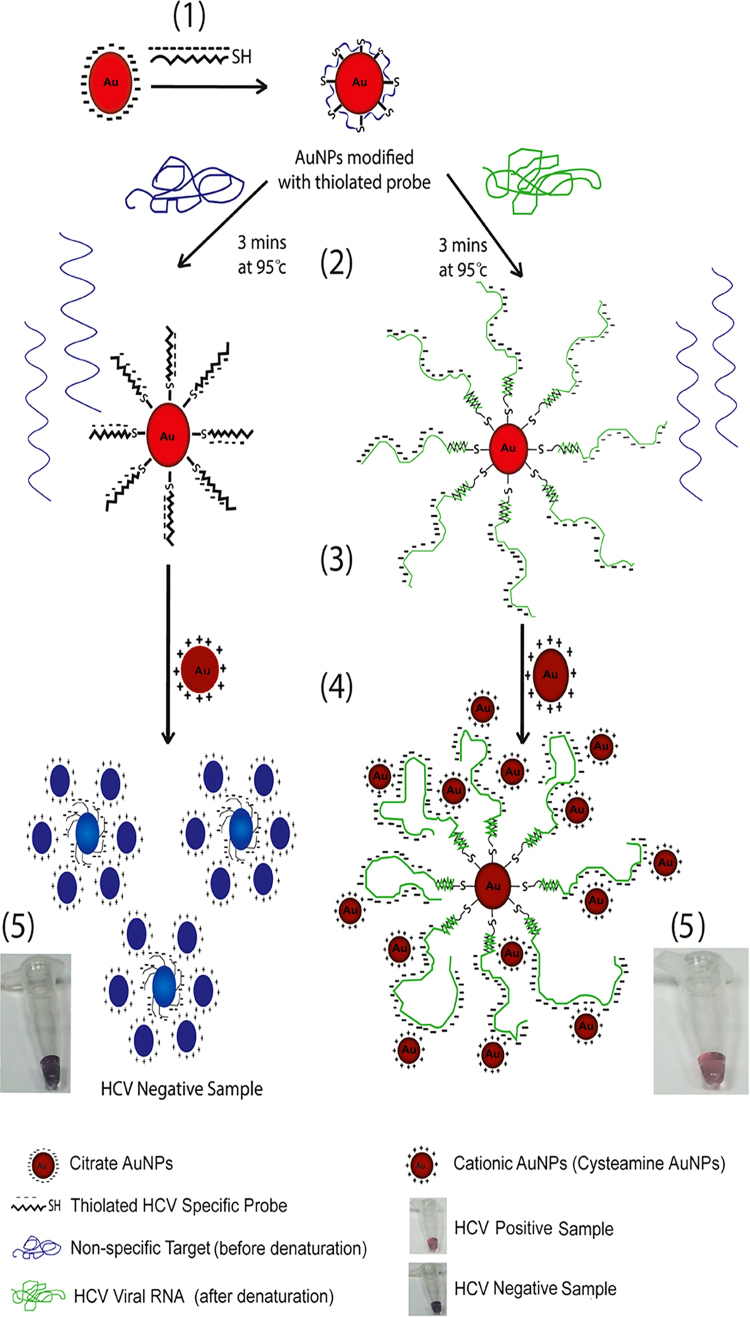
A scheme depicting the nano-assay principle and procedures. (1) Citrate capped AuNPs, was functionalized with thiolated HCV specific probe forming nanoprobes. (2) The nanoprobe is then mixed with the RNA sample and heated at 95 °C for 3 min. (3) *Right panel*; the HCV viral RNA was hybridized to the HCV specific nanoprobe by sequence complimentarily. *Left panel*; no HCV RNA is present (non-specific RNA target), thus no hybridization takes place. The mixture incubated at room temperature. (4) The cationic AuNPs was added. (5) *Right panel;* in the presence of HCV RNA, the mixture solution remains red, reflecting the dispersion of the AuNPs onto the folded HCV RNA, thereby protecting the nanoprobes from aggregation by cationic AuNPs. *Left panel;* in the absence of a complementary target, the cationic AuNPs will bind to the probe phosphate backbone electrostatically, thereby reducing the inter-particle distance between the nanoprobes and the cationic AuNPs, resulting in aggregation and color change from red to blue. (For interpretation of the references to color in this figure legend, the reader is referred to the web version of this article).

Herein, we utilized positively charged gold nanoparticles (cysteamine and CTAB AuNPs) to induce aggregation of citrate capped AuNPs decorated with an HCV RNA specific probe (Graphical abstract and Fig. 1). Our aim was to minimize the factors that may affect the output results, enhance the specificity, sensitivity and detection limit. Most importantly, this approach allowed us to achieve quantitative detection of HCV RNA in clinical samples, in short time and with reasonable cost, and it could be easily adapted for full automation. Furthermore, the nano-assay was validated using other RNA transcripts extracted from cell lines to illustrate its broad utility as a nucleic acid detection technology.

## 2 Materials and methods

### 2.1 Chemicals and equipments

Hydrogen tetracloroaurate (III) trihydrate (HAuCl_4_·3H_2_O) ˃99%, Dithiothrietol, Dibasic and mono basic phosphate, Sodium dodecyl sulfate (SDS), Sodium Chloride, Sodium borohydride, Hexadecyltrimethyl ammonium bromide (CTAB), Tri-sodium citrate dehydrate, and Ribonuclease A were purchased from Sigma- Aldrich. Cysteamine (2-Mercaptoethylemine HCl ˃98% was purchased from Acros Organics). SV-Total RNA isolation system was purchased from Promega. Artus HCV RG RT-PCR Kit and QIAmp viral RNA kit were purchased from Qiagen. RPMI-1640 medium, L-glutamine, Penicillin/streptomycin was purchased from Lonza, while fetal bovine serum from Gibco. NAP-5 columns (illustra NAP-5, GE Healthcare).

UV–vis spectra were recorded with Eppendorf Bio-spectrophotometer basic. A zetasizer ZC system (Malvern Instrument Ltd., Zeta sizer Nano series, UK) was used for size and potential measurements. High resolution transmission electron microscope (HRTEM, JEM-2100) was used for imaging

### 2.2 Serum samples collection

Sample Collection was done following ethical approval and informed consent of all subjects. HCV infected serum samples were collected (n=28), which included 23 samples with chronic HCV infection and 5 samples with acute HCV infection. Healthy volunteers provided 17 serum samples. Rapid HCV and Hepatitis B virus (HBV) antibody tests were performed for all samples. Only one sample was HBV positive. Real-time PCR was conducted for all the samples using Artus HCV RG RT-PCR Kit according to the manufacturers’ instructions.

### 2.3 Synthesis, functionalization and characterization of citrate AuNPs and nanoprobes

Citrate AuNPs was prepared by the traditional sodium citrate reduction method of Gold (III) chloride (Hill and Mirkin, 2006, Turkevich, 1985a, Turkevich, 1985b). Characterization was done using Transmission Electron Microscope (TEM), UV–Vis. spectroscopy and Dynamic light scattering (DLS). Citrate AuNPs were functionalized with each alkanethiol modified RNA target specific probe using well known salt aging process (Hurst et al., 2006). Functionalization of the alkanethiol probes was done exactly as described (Hill and Mirkin, 2006). HCV specific probe is an antisense primer of 32 bases complementary to the highly conserved region in the HCV RNA 5′-untranslated region (5′UTR) in almost all genotypes and subtypes. The probe (5′-TACCACAAGGCCTTTCGCGACCCAACACTACT’-3) was alkane-thiol modified at its 5′-terminus. In parallel reactions, functionalization was performed for Topoisomerase 1&2 (TOP1 and & TOP2), Tyrosyl- DNA phosphodiesterases 1&2 (TDP1 and TDP2) transcripts, by alkanethiol specific probes for each transcript extracted from Rectal Cancer carcinoma (RKOs) cell lines. Each transcript specific probe was functionalized to the AuNPs solution separately.

### 2.4 Synthesis and characterization of Cysteamine AuNPs

Cysteamine AuNPs were synthesized using sodium borohydride reduction method as described (Kim, Kim et al., 2009). Cysteamine AuNPs was characterized using TEM, DLS, and spectrophotometrically.

### 2.5 Synthesis and characterization of CTAB AuNPs

CTAB AuNPs synthesis is generally performed following the established seed-mediated growth using two solutions (El-Sayed et al., 2006, Huang et al., 2007b, Li et al., 2013, Scarabelli et al., 2015). Herein, CTAB AuNPs was synthesized using only the seed solution in a single-phase reaction without the growth solution. In a typical experiment, 3.7 ml of 0.2 M aqueous solution of CTAB was mixed with 20 µl of 1 mM gold chloride under vigorous stirring at 50 °C. Then, 1 ml of ice-cold 10 mM sodium borohydride was added in three portions with 20 min intervals. Solution color was changed from yellow to light brown after the first addition of the borohydride and color intensity increased after each addition. Stirring was continued for 3 h at 50 °C and then stored at room temperature for 2 days at dark. A clear pink-colored solution was obtained after two days, which is followed by characterization as described above for AuNPs.

### 2.6 HCV RNA extraction evaluation

HCV RNA extraction was performed using Promega SV-total RNA isolation system according to the modified HCV extraction manufacturer's protocol (Otto et al., 1998). The QI-Amp Viral RNA kit was used for HCV RNA extraction to select the best RNA extraction kit compatible with the nano-assay.

### 2.7 Cell lines and total RNA extraction

RKOs cancer cell lines (Homo sapiens, Tissue: colon, Carcinoma) were cultured at 37 °C and 5% CO_2_ in RPMI-1640 medium, supplemented with 10% fetal bovine serum, 1% L-glutamine and 1% penicillin/streptomycin. Total RNA extraction was conducted using Promega SV-total RNA isolation system following manufacture instructions and each transcript was further purified by homemade magnetic nanoparticles as described previously (Eissa et al., 2014, Shawky et al., 2014), to enhance the purity of the extracted transcript.

### 2.8 Colorimetric/spectrophotometric AuNPs assay for RNA detection

The assay was performed by mixing 5 µl of nanoprobe with 10 µl of RNA sample, heated at 95 °C for 3 min, incubated at room temperature for 5–10 min. Cationic AuNPs (10 µl) was then added to the solution and mixed well. Solution color was developed immediately and observed by naked eye while mixing. The color was stable for ~30 min, depending on the RNA concentration. Solutions were scanned from 400 nm to 750 nm using spectrophotometer. To confirm that the color change is due to RNA solely, the nano-assay was performed on two HCV positive samples before and after digestion with Ribonuclease A enzyme. Each sample was divided into two portions, one portion was treated with the enzyme by adding 10 µl of 10 mg/ml Ribonuclease A to 35 µl of HCV RNA and then incubated for 20 min at room temperature. After completion of the digestion process, the assay was performed on the Ribonuclease A treated sample and on the mock-treated sample. The color of the two samples was then observed and samples were also analyzed using TEM.

### 2.9 Quantification of HCV RNA using real-time RT-PCR and the developed nano-assay

HCV RNA viral load was determined using Artus Qiagen HCV Real-time RT-PCR Kit, according to the manufacturer manual, for all sera samples. Quantification of HCV RNA using the nano-assay was performed by preparing serial dilutions of HCV RNA concentration (10–1200 IU/µl), and examined using the nano-assay. The spectral absorbance for each concentration was scanned spectrophotometrically in duplicate, and the ratio of the non-aggregated nanoparticles at λ530 to the aggregated nanoparticles at λ_650_ (A_530_/ A_650_) was recorded and used to generate the standard curve, in which the A_530_/ A_650_ ratio was plotted against the viral RNA log concentration. All HCV RNA samples viral load were determined by the nano-assay using the generated standard curve, from their respective A_530_/ A_650_ ratios, using the equation generated by the standard curve.. The results were expressed in IU/µl and converted to IU/ml by (IU/µl *RNA elution volume in µl/ serum volume in µl**)**. Comparison between the developed nano-assay and the Real-Time RT-PCR viral load is shown in Table S1, and Fig. 6c–f. Receiver operating characteristic curve (ROC curve) for the nano-assay was generated to determine the specificity, sensitivity, and the detection limit (Table S2), using SPSS software (IBM, SPSS Statistics, version 20 package). Detection limit was further confirmed by performing serial dilutions of HCV sample till 1 IU/µl. Each dilution was tested by the nano-assay until aggregation occurred starting from about 4 IU/µl.

### 2.10 Nano-assay TEM analysis for positive, and HCV negative samples, and Ribonuclease A treated sample before and after digestion

TEM analysis was conducted immediately after performing the nano-assay for both HCV positive and negative samples. Moreover, it was performed on the Ribonuclease treated and mock-treated sample as described in Section 2.8 to verify that RNA folding contributes to nanoprobe stability.

## 3 Results and discussion

### 3.1 Citrate AuNPs synthesis, functionalization and characterization

**Fig. 2:**
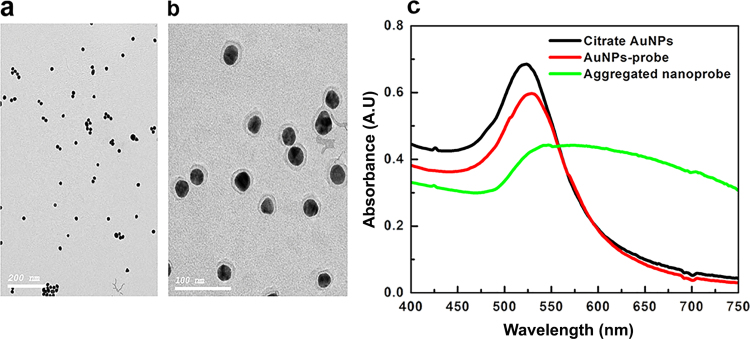
Characterization of citrate-capped AuNPs. (a) A representative TEM image of the citrate-capped AuNPs (b) and of the nano-probe (c). The spectra of the citrate capped AuNPs showing SPR λ_max_ at approximately 520 nm. The nanoprobe showed a slight red shift to 530 nm with a decrease in the peak intensity indicating functionalization. Cysteamine AuNPs induced aggregation of the nanoprobe spectrum was shown by brooding in its peak and the lack of SPR.

Citrate AuNPs morphology were characterized by TEM analysis. As-synthesized AuNPs were spherical and uniformly distributed with average diameter of ~20 nm (Fig. 2a) and average charge of −48 mv. Size and the charge were further measured using DLS (Fig. S1a and b). HCV nanoprobe was synthesized by functionalization of the Citrate AuNPs with the alkanethiol modified probe. Functionalization exploits the thiol group, which possesses a higher affinity to the gold surface than citrate, thereby forming a strong covalent bond (Xue et al., 2014), providing high stability to the nanoprobe against high salt, and positive ligands concentrations induced aggregation. TEM analysis of HCV nanoprobe revealed an increase in the citrate AuNPs from ~20 nm to ~38 nm (Fig. 2b), which was confirmed by DLS with a corresponding reduction of surface charge from −48 mv to −27 mV (Fig. S2a and b). Spectrum of the as-synthesized AuNPs revealed a λ_max_ at 520 nm, while that of the nanoprobe showed a slight red shift to 530 nm (Fig. 2c). The red shift was accompanied by a noticeable absorption broadening upon cysteamine AuNPs induced aggregation of the nanoprobe (Fig. 2c). Molar concentration of AuNPs was calculated to be 4 nM with ~2.436×10^12^ nanoparticles per ml (Liu et al., 2007). Together, these data demonstrate proper conjugation of probes to the particles and confirm the ability of cysteamine AuNPs to induce aggregation of the nanoprobe.

The probe amount and density conjugated to citrate AuNPs are crucial for downstream applications. At high amount, steric hindrance at the AuNPs surface takes place, resulting in electrostatic repulsion between the target and the probe, and thus no hybridization takes place (Doria et al., 2010). It was estimated that ~1.44 nmol probes were conjugated to the AuNPs. Accordingly, the amount of probes per one nanoparticle was ~120 oligonucleotides, which is in agreement with previously reported functionalization methods (Hurst et al., 2006).

### 3.2 Cysteamine and CTAB AuNPs synthesis and characterization

**Fig. 3:**
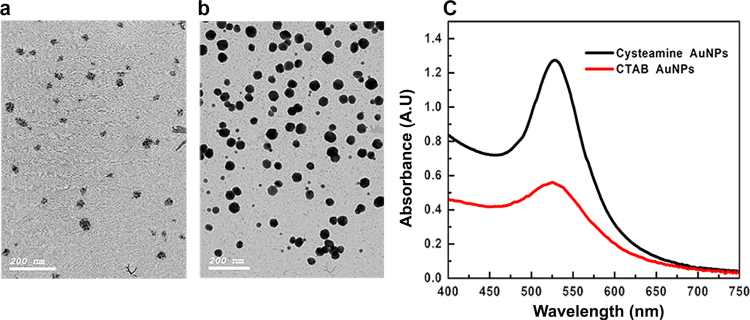
Characterization of CTAB and Cysteamine AuNPs. (a) TEM image of CTAB AuNPs showing the nanoparticles appearing as clusters of small AuNPs surrounded or internalized within a shell. (b) TEM image showing Cysteamine AuNPs. (c) The extinction spectra of the cysteamine AuNPs and CTAB AuNPs shows λ_max_ of 528 and 526 nm; respectively.

CTAB and cysteamine AuNPs morphology were characterized using TEM (Fig. 3a and b, respectively). Cysteamine AuNPs was spherical and uniformly distributed with an approximate size of 40 nm and a λ_max_ of ~528 nm (Fig. 3c), with an average positive charge of +39. The uniformity and charge were further assessed by DLS (Fig. S3a and b). The Molar concentration was calculated as described (Liu et al., 2007) and found to be ~0.5 nM with ~3.04×10^11^ nanoparticles per ml. The charge and size of CTAB AuNPs were +100 mv and 30 nm, respectively (Fig. S4a and b). CTAB AuNPs extinction spectrum revealed one sharp peak in the visible region with λ_max_ at 526 nm, suggesting spherical nanoparticles with uniform size (Fig. 3c). However, their TEM analysis (Fig. 3a) showed core/shell like structure of many AuNPs coated within a shell. This is may be due to AuNPs seeds formation followed by their internalization within the CTAB micelle. We propose that synthesis of CATB AuNPs from seed solution only is primarily affected by reagent concentrations, especially the reductant to the gold precursor, which was increased by ~450 times, and the CTAB which was increased by two times, compared to conventional methods (Orendorff and Murphy, 2006). Once the seeds are formed in the presence of the high concentration of CTAB and reaction high temperature that stabilized the CTAB micelles and preserved the particles from aggregation, it led to extra layers on the nanoparticles, which appeared as a shell like structure (Fig. 3a). Moreover, the addition rate of the reductant (3 times with 1 h intervals) and its high concentration led to complete reduction of all the gold ion states to gold metal. Indeed, the high reaction temperature affects the final AuNPs shape and size (Scarabelli et al., 2015). Thus, the high temperature employed here and long growth time (2 days) led to further growth of the traditional seed particles into bigger nanoparticles and prevented the CTAB molecules from crystallization.

### 3.3 Selection of cysteamine AuNPs for the nano-assay

**Fig. 4:**
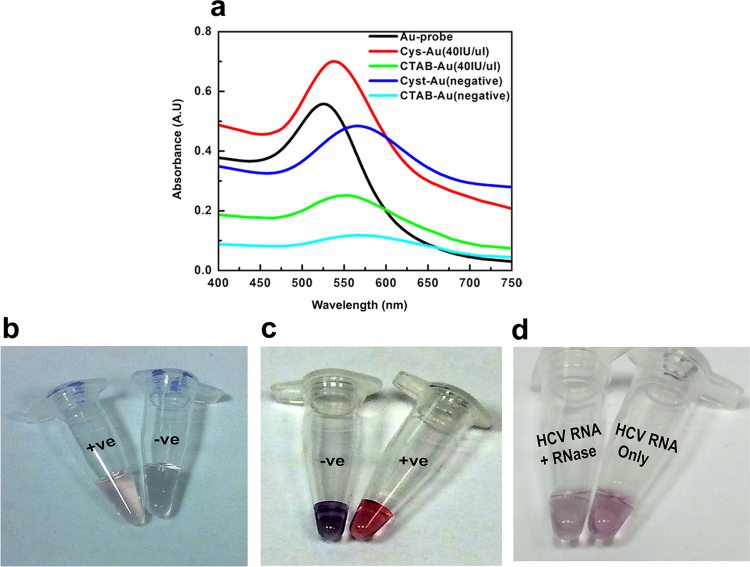
Analyses of HCV clinical samples using cysteamine and CTAB AuNPs. (a) Extinction spectra of positive and negative HCV samples detected by the cysteamine and CTAB AuNPs. *Note the difference in the peak intensity between the two cationic AuNPs used*. Cysteamine AuNPs showed better SPR and well defined peak than the CTAB AuNPs. In contrast to CTAB AuNPs, no significant difference between the SPR of cysteamine AuNPs in the positive sample compared to the nanoprobe peak was observed. (b) A representative photograph showing the change in color using CTAB AuNPs biosensors. We note the faint colors of both the positive and negative samples. (c) A representative photograph showing the change in color using cysteamine AuNPs biosensors. Note the brighter colors produced compared to CTAB AuNPs biosensors. (d) The assay was performed on HCV positive sample with and without the addition of Ribonuclease A (RNase A). While the tube on the right showed faint red color indicating the presence of HCV RNA, the faint color is due to the sample was diluted before photographing, for proper comparing with the other tube. The RNase A treated tube on the left was nearly colorless indicating the absence of RNA. The difference in color confirms that the HCV RNA accounts for the stability of the AuNPs solution. (For interpretation of the references to color in this figure legend, the reader is referred to the web version of this article).

We noticed higher degree of aggregation and broader spectra of CTAB-AuNPs than cysteamine-AuNPs when incubated with either HCV RNA or control samples (Fig. 4a). Spectral behavior of CTAB AuNPs on HCV positive samples was closely similar to that produced with monovalent or divalent cations in the conventional non-cross linking assays (Conde et al., 2010). This is likely due to the highly stable positively charged quaternary ammonium (NH_4_^+^) cation, which ultimately increases false negative possibilities. Therefore, we decided to use cysteamine-AuNPs that are capable of acting as hydrogen bond donors/acceptors towards the nanoprobe-HCV RNA. This also led to improved color detection since CTAB AuNPs colors were fainter (Fig. 4b) compared to cysteamine AuNPs (Fig. 4c) in both positive and negative samples.

### 3.4 Evaluation of different RNA extraction Kits

Total RNA Promega Kit was chosen for RNA extraction. Selection of Promega Kit over the Qiagen viral RNA extraction kit was based on that the high rate of false positives recovered with the latter. This is likely due to DNA contamination hence the kit was not designed to separate viral RNA from cellular DNA (Qiagen, 2014). The presence of circulating cell free DNA in the serum with high concentration along with some genomic DNA in the final product had negative impact on assay performance, masking the nanoprobe from aggregation and increases false positive outcomes. In contrast, Promega total RNA isolation kit includes a DNase treatment step, which eliminates DNA from the final product, providing pure RNA with reproducible results.

### 3.5 Factors affecting nano-assay performance

The nano-assay reproducibility, sensitivity and specificity are primarily affected by (1) nanoprobe and cationic AuNPs concentration and type, (2) pH of the reaction mixture, (3) temperature and (4) RNA folding. The nanoprobe density of ~120 probes per small sized AuNPs ~20 nm is known to improve its stability against aggregation and provides acceptable amount for proper probe/RNA hybridization (Hurst, Lytton-Jean et al., 2006). Final nanoprobe and cationic AuNPs concentrations greatly affected the nano-assay final results. The expected outcome was observed when CATB AuNPs concentration was less than nanoprobe concentration. However, higher concentration of CTAB AuNPs caused nanoprobes aggregation even in the presence of the RNA, likely due to the CTAB strong positively charged quaternary ammonium cation. In contrast, cysteamine AuNPs gave superior results regardless of the cysteamine AuNPs or nanoprobe concentration. The best results obtained when cysteamine AuNPs was twice the nanoprobe amount. Moreover, the spectrum of samples following cysteamine AuNPs had sharp peaks and showed a clear difference between the positive and negative samples. This allowed a linear relation to be devised and thus enabled quantitative detection of RNA. In contrast, CTAB AuNPs spectra were not clear enough to differentiate between different RNA concentrations and thus couldn't be used for quantitation. Comparison between the two cationic AuNPs regarding the absorption peaks and color intensity of the nano- assay is shown in Fig. 4a–c. Key to this enhanced performance is most likely that cysteamine short molecular dimension, which allowed better distribution on the folded HCV RNA backbone.

Solution pH had no impact on the nano-assay as the CTAB AuNPs quaternary ammonium group is always positively charged regardless of the pH while the cysteamine protonated amino group positive charge predominates from pH 9.3, which is way above the assay pH at 7.4. The non-pH dependency of our assay is superior to the non-cross linking assays described previously where pH is a main player for assay performance, especially in the target/mismatched probes (Sato et al., 2005). Therefore, using cationic AuNPs excludes the pH parameter from the assay. The last two parameters affecting assay performance are reaction temperature and nucleic acid folding. Reaction temperature highly affected the nano-assay. Immediate aggregation has occurred upon mixing the nanoprobe with the cationic AuNPs even with very high RNA concentrations. This is due to electrostatic interaction affinity between the probes’ nitrogenous bases and the citrate AuNPs, which prevents hybridization between the RNA target and the probes (Li and Rothberg 2004b). A brief denaturation step was therefore conducted for the nanoprobe/RNA mixture and prior to the addition of the cationic AuNPs to allow accessibility of the probes to the RNA molecules (Larguinho et al., 2015), and thus allowing for the unfolding of the RNA tertiary structure and subsequent hybridization with its complementary probes. Incubation of the mixture at room temperature prior to the addition of the cationic AuNPs was essential. The time/temperature gap between heating and the addition of cationic AuNPs allowed for RNA hybridization to the probe and subsequent RNA re-folding to adopt its most thermodynamically stable form (Doerrbecker et al., 2013, El Awady et al., 2009).

**Fig. 5:**
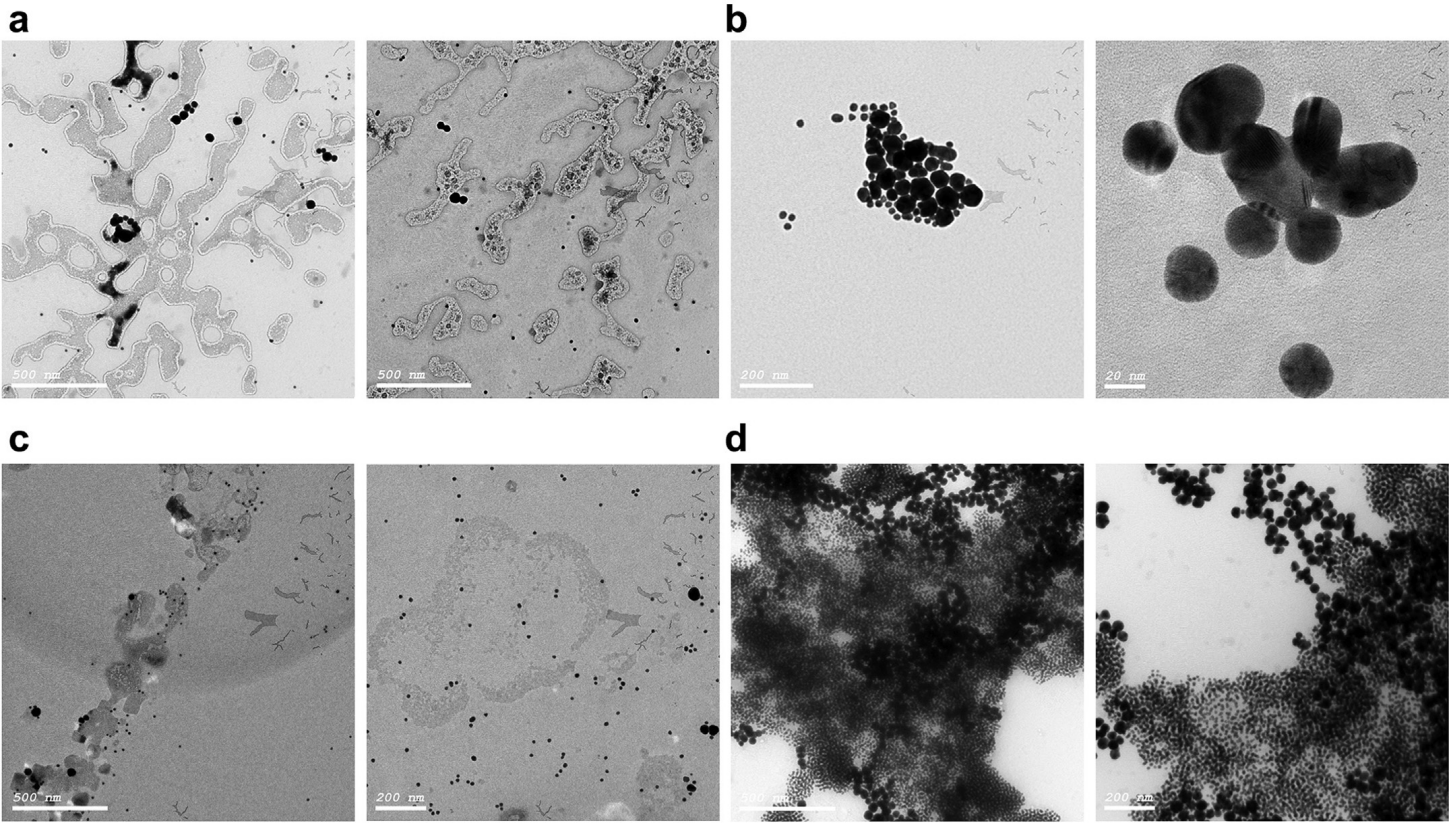
Characterization of AuNPs in the presence of HCV RNA. (a) Representative TEM images for HCV positive samples after performing the assay showing the distribution of the nanoparticles (black spheres) along and within the RNA molecules and in solution. The RNA folding protects the nanoprobe from aggregation and provides a large surface area for the cationic AuNPs to be distributed on. (b) Representative TEM images showing the aggregation of the nanoparticles in absence of HCV RNA. The inter-particle distance between the AuNPs decreases and thus induces aggregation. (c) TEM images for another HCV positive sample after performing the assay, which is consistent with images in panel a. (d) The same sample was subjected to the developed nano-assay after treatment with Ribonuclease A. *Note that digestion of RNA molecules caused aggregation of the AuNPs with some residues of RNA whereas the RNA negative sample also showed aggregation with no residual RNA*.

We propose RNA folding adaptation to coat the nanoprobe after hybridization, resulting in multiple layers of RNA, thereby stabilizing the nanoprobe. The shape adopted by the re-folded RNA is predicted to give high surface area for the cationic AuNPs to be distributed along the RNA molecules, preventing its interaction with the nanoprobe and preserving their distribution. This notion was confirmed by comparing TEM results for HCV positive and negative samples (Fig. 5a and b). To further validate the assay, HCV samples were re-analyzed with and without prior digestion with RNase A. In RNase A treated samples, aggregation of the AuNPs occurred, as was the case for negative samples, indicating absence of RNA. In contrast, in mock treated samples the stability of AuNPs was noticeable with a detectable red color (Fig. 4d), which was further confirmed by TEM (Fig. 5c and d).

### 3.6 Quantitative determination of HCV RNA in serum samples using the nano-assay

As the concentration of the RNA increased the nanoprobe λ_max_ height at wavelength 530 nm proportionally increased, representing the non-aggregated AuNPs (dispersed nanoparticles, Fig. S6a). The opposite occurred as the RNA concentration decreased, the nanoprobe λ_max_ height declined with concomitant shift and broadness in the peak to a higher wavelength, indicating an increase in the aggregated AuNPs population (non-dispersed AuNPs). Thus, at high RNA concentration the ratio of the non-aggregated/aggregated is high, indicating the predominance of the dispersed nanoprobes and their stability and vice versa. The standard curve (Fig. S6b) was plotted by recording the spectral absorbance at λ_530_/λ_650_ ratio against RNA log concentration. The plot showed a linear relation (Y=1.1143x+1.1066, R^2^=0.97). All sample concentrations were calculated using the standard curve and ratio described above. Also, some samples with different concentrations were plotted on the standard curve in Fig. S7, and a linear relation has been obtained (Y=1.2008x+1.0055, R^2^=0.966). All samples quantified by the nano-assay were compared to the gold standard Real-Time RT-PCR method for HCV quantification (Table S1). The Real-Time RT-PCR revealed 27 out of the 28 as positive HCV samples, whereas the nano-assay revealed 26 out of 28 as positive with a concordance of ~96.3%. Three out of the 17 HCV-negative samples were detected as HCV-positive by the qRT-PCR. Surprisingly, the same three samples were detected as HCV-positive by the nano-assay so the concordance with the qRT-PCR in the negative samples was 100%. These three samples were negative by an HCV RNA rapid antibody detection test but were found positive by an ELISA assay. Moreover, HCV anti-IgM was negative and consequently these three samples were classified in this study as asymptomatic chronically HCV infected. After including the three samples as HCV positive the assay specificity increased to 100% instead of 94.1% and sensitivity increased to 93.3% instead of 89.3% at detection limit 4.57 IU/µl.

### 3.7 Charge and size of different AuNPs and comparison between real-time RT-PCR and the nano-assay

**Fig. 6:**
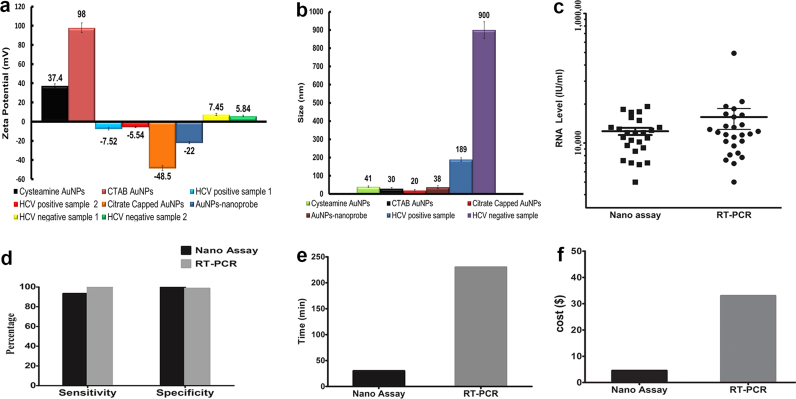
Summary of the size and zeta potential, and a comparison between the Real Time RT-PCR and the nano-assay. (a) Charges of all AuNPs used in this study. HCV negative samples have positive charge while HCV positive samples have negative charge. (b) The size of the different AuNPs preparations and the HCV positive & negative samples. A significant size increase for the negative samples was observed that confirms the aggregation of the nanoparticles. The mild increase in size of the positive sample may be due to a slight aggregation of some nanoparticles in the reaction mixture, which is not significant as the color of the sample did not change and was dependent on RNA concentration. (c) The mean viral loads (IU/ml) and standard deviations for the samples tested using the nano-assay (15203±1898) and the RT-PCR (15203±1898) respectively, showing no significant difference in the final viral load between the two methods. (d) The nano-assay showed a sensitivity and specificity of 93.3% and 100%, while RT-PCR showed a **96.8% sensitivity and 100% specificity** . (e) The overall time of the nano-assay for one sample was ~30 min compared to 230 min for the RT PCR (f) Cost per sample for the developed biosensor and the RT PCR was 4.5 and 33 USD, respectively; cost including all materials, chemicals and plastics used for the assays, and the RNA extraction cost. (For interpretation of the references to color in this figure legend, the reader is referred to the web version of this article).

HCV positive samples charge was negative indicating the distribution and spreading of cationic AuNPs along the RNA molecules, thereby preventing the aggregation of the nanoprobe (Fig. 6a and S5a, right panel). In contrast, HCV negative samples (aggregated AuNPs) were positively charged indicating the alignment of the cationic AuNPs onto the nanoprobe phosphate backbone, and reduction of the inter-particle distance between the nanoprobes and the cationic AuNPs (Fig. 6a and S5b, right panel). It was associated with a reduction in inter-particle distance leading to increase in the size to ~900 nm (Fig. 6b and S5b, left panel). However, the HCV positive sample size increased to ~190 nm (Fig. 6b and S5a, left panel), which is likely due to differences in the amount of the dispersed to non-dispersed AuNPs, which depends mainly on the RNA concentration of the tested sample. Finally, we examined whether the nano-assay could also be applied to other RNAs from different sources (e.g. cell lines). Four RNA transcripts implicated in the repair of protein-linked DNA breaks were tested using specific nanoprobes for each transcript (Ashour et al., 2015, Elsayed et al., 2016, Meisenberg et al., 2016). In agreement with HCV detection, the results of the additional four transcripts confirm the broad utility of the biosensor technology in RNA detection (Fig. S8). A summary of the size and charge of nanoparticles, and a comparison between the Real Time RT-PCR and the nano-assay are depicted in Fig. 6.

## 4 Conclusions

We developed a novel AuNPs quantitative biosensor colorimetric assay for the direct detection of unamplified HCV RNA in clinical samples and DNA repair transcripts from cell lines. Stability or aggregations of the nanoprobes rely on cationic AuNPs and RNA target folding rather than the medium ionic strength or pH, which limits false positives. The assay is simple with a turnover time of ~30 min including RNA extraction, sensitive, specific, cost effective and could readily be adopted for full automation.

## Footnotes

Appendix A Supplementary data associated with this article can be found in the online version at doi:10.1016/j.bios.2016.11.001.

## References

[bib1] AASLD-IDSA, 2016. Recommendations for testing, managing, and treating hepatitis C. AASLD-IDSA, USA. 〈http://www.hcvguidelines.org/full-report/hcv-testing-and-linkage-care〉 accessed, May 2016.

[bib2] Ashour M.E., Atteya R., El-Khamisy S.F. (2015). Nat. Rev. Cancer.

[bib3] Atrah H.I., Ahmed M.M. (1996). J. Clin. Pathol..

[bib4] Baptista P., Pereira E., Eaton P., Doria G., Miranda A., Gomes I., Quaresma P., Franco R. (2008). Anal. Bioanal. Chem..

[bib5] Cloherty G., Talal A., Coller K., Steinhart C., Hackett J., Dawson G., Rockstroh J., Feld J. (2016). J. Clin. Microbiol..

[bib6] Conde J., de la Fuente J.M., Baptista P.V. (2010). J. Nanobiotechnol..

[bib7] Doerrbecker J., Meuleman P., Kang J., Riebesehl N., Wilhelm C., Friesland M., Pfaender S., Steinmann J., Pietschmann T., Steinmann E. (2013). J. Viral Hepat..

[bib8] Doria G., Baumgartner B.G., Franco R., Baptista P.V. (2010). Colloids Surf. B Biointerfaces.

[bib9] Eissa S., Shawky S.M., Matboli M., Mohamed S., Azzazy H.M. (2014). Clin. Biochem..

[bib10] El Awady M.K., Azzazy H.M., Fahmy A.M., Shawky S.M., Badreldin N.G., Yossef S.S., Omran M.H., Zekri A.R., Goueli S.A. (2009). World J. Gastroenterol..

[bib11] Elghanian R., Storhoff J.J., Mucic R.C., Letsinger R.L., Mirkin C.A. (1997). Science.

[bib12] El-Sayed I.H., Huang X., El-Sayed M.A. (2006). Cancer Lett..

[bib13] Elsayed W., El-Shafie L., Hassan M.K., Farag M.A., El-Khamisy S.F. (2016). Isoeugenol is a selective potentiator of camptothecin cytotoxicity in vertebrate cells lacking TDP1. Scientific Reports.

[bib14] Hajarizadeh B., Grebely J., Dore G.J. (2013). Nat. Rev. Gastroenterol. Hepatol..

[bib15] Hill H.D., Mirkin C.A. (2006). Nat. Protoc..

[bib16] Huang K.S., Lin Y.C., Su K.C., Chen H.Y. (2007). Biomed. Microdevices.

[bib17] Huang X., Jain P.K., El-Sayed I.H., El-Sayed M.A. (2007). Nanomedicine.

[bib18] Hurst S.J., Lytton-Jean A.K., Mirkin C.A. (2006). Anal. Chem..

[bib19] Kim J.W., Kim J.H., Chung S.J., Chung B.H. (2009). Analyst.

[bib20] Larguinho M., Canto R., Cordeiro M., Pedrosa P., Fortuna A., Vinhas R., Baptista P.V. (2015). Expert Rev. Mol. Diagn..

[bib21] Lee J., Conniff J., Kraus C., Schrager S. (2015). Wis. Med. J..

[bib22] Li C.M., Zhen S.J., Wang J., Li Y.F., Huang C.Z. (2013). Biosens. Bioelectron..

[bib23] Li H., Rothberg L. (2004). Proc. Natl. Acad. Sci. USA.

[bib24] Li H., Rothberg L.J. (2004). J. Am. Chem. Soc..

[bib25] Li H., Rothberg L. (2005). Anal. Chem..

[bib26] Liandris E., Gazouli M., Andreadou M., Comor M., Abazovic N., Sechi L.A., Ikonomopoulos J. (2009). J. Microbiol. Methods.

[bib27] Liu X., Atwater M., Wang J., Huo Q. (2007). Colloids Surf. B Biointerfaces.

[bib28] Mauss S., Berg J.R., Rockstroch J., Sarrazin C., Wedemyer H. (2013). Hepatology.

[bib29] Meisenberg C., Ashour M.E., El-Shafie L., Liao C., Hodgson A., Pilborough A. (2016). Epigenetic changes in histone acetylation underpin resistance to the topoisomerase I inhibitor irinotecan. Nucleic Acids Research.

[bib30] Miller F.D., Abu-Raddad L.J. (2010). Proc. Natl. Acad. Sci. USA.

[bib31] Orendorff C.J., Murphy C.J. (2006). J. Phys. Chem. B.

[bib32] Paul Otto, Dan Kephart, Rex Bitner, Suzanne Huber, Volkerding, K., 1998. Separate Isolation of Genomic DNA and Total RNA from Single Samples Using the SV Total RNA. promega notes, p. 6.

[bib33] Qiagen (2014). QIAamp® Viral RNA Mini Handbook.

[bib34] Sato K., Hosokawa K., Maeda M. (2003). J. Am. Chem. Soc..

[bib35] Sato K., Hosokawa K., Maeda M. (2005). Nucleic Acids Res..

[bib36] Scarabelli L., Sanchez A., Perez-Juste J., Liz-Marzan L.M. (2015). J. Phys. Chem. Lett..

[bib37] Scott J.D., Gretch D.R. (2007). J. Am. Med. Assoc..

[bib38] Seifert L.L., Perumpail R.B., Ahmed A. (2015). World J. Hepatol..

[bib39] Shawky S.M., Guirgis B.S., Azzazy H.M. (2014). Clin. Chem. Lab. Med..

[bib40] Turkevich J. (1985). Gold Bull..

[bib41] Turkevich J. (1985). Gold Bull..

[bib42] WHO, 2016. Hepatitis C, Fact N164, 〈http://www.who.int/mediacentre/factsheets/fs164/en/〉 accessed, March 2016.

[bib43] Xue Y., Li X., Li H., Zhang W. (2014). Nat. Commun..

