# Supplementary material for: Gold aggregating gold: A novel nanoparticle biosensor approach for the direct quantification of hepatitis C virus RNA in clinical samples
Source: Biosens Bioelectron. 2017 Jun 15;92:349–56. doi: 10.1016/j.bios.2016.11.001 (PMC5345390; doi:10.1016/j.bios.2016.11.001)
Supplement: Supplementary file 1 — Supplementary material [file mmc1.pdf]

Supplementary Materials

Gold Aggregating Gold: A Nanoparticle Biosensor Approach for the Direct Quantification of Hepatitis C Virus RNA in Clinical Samples

Sherif M. Shawky[1,2,3], Ahmed A. Awad[1], Walaa Allam[1], Mohamed H. Alkordi[4], and Sherif F. EL-Khamisy[1,2*]

[1]Center of Genomics, Helmy institute, Zewail City of Science and Technology, Sheikh Zayed Dist. 12588, Giza, Egypt

[2]Krebs Institute, Department of Molecular Biology and Biotechnology, University of Sheffield, Sheffield, S10 2TN, UK

[3]Misr University for Science and Technology, Faculty of Pharmacy, Biochemistry Department, Giza, Egypt

[4]Center for Materials Science, Zewail City of Science and Technology, Sheikh Zayed Dist. 12588, Giza, Egypt

*To whom correspondence should be addressed: Email: s.el-khamisy@sheffield.ac.uk, Tel: +44 114 2222 791; Fax: +44 114 2222 2850

Experimental

1. Molar concentration calculations of the synthesized AuNPs nanoparticles

Molar concentration of the AuNPs was calculated using equations A.1 and A. 2, assuming that all the gold ions have been consumed in the reaction and the particles are spherical in shape forming face-centered cubic (FCC) phase within the AuNPs (Liu et al. 2007)

$$N = \frac{\pi}{6}\frac{\rho D^3}{M} = 30.89602\ D^3 \quad \textbf{(Equation A.1)}$$

Where: **N** is the average number of gold atoms per particle, $\boldsymbol{\rho}$ is the density for fcc gold (19.3 g/cm3), **M** is the atomic weight of gold (197 g/mole), and **D** is the average diameter, in nanometer, for the AuNPs as calculated from the zetasizer measurements.

Then, the molar concentration is calculated according to this equation:

$$C = \frac{N_{Total}}{NVN_A} \quad \textbf{(Equation A.2)}$$

In which: the $\mathbf{N_{total}}$ is the initial moles of gold ions used in the reaction, **N** is the average number of gold atoms calculated from equation 1, while **V** is the volume of the reaction solution in liters, and $\mathbf{N_A}$ is Avogadro's number.

The above calculations were used for the cysteamine AuNPs solutions.

2. Functionalization of citrate AuNPs with RNA target-specific probe (nanoprobe)

Functionalization of the citrate capped AuNPs was performed by salt aging process (Hurst et al. 2006). Briefly, 5 nmol of alkanethiol-functionalized RNA specific probe (thiolated probe) was first lyophilized, and then, the disulfide cleavage of the probe was achieved by re-suspension the freshly lyophilized probe in 100 µl of 0.1 M dithiothrietol, dissolved in 0.17

M phosphate buffer (PH=8), for 2 to 3 hours with occasional vortexing, while wrapped in foil. Purification of the cleaved probes was done using NAP-5 columns, according to the manufacturer's instructions. The concentration after cleavage was measured using UV-Visible spectrophotometer at 260 nm using Beer's Lambert Law. Freshly cleaved probes concentrations were calculated at λ_{260}, and then were added to the gold nanoparticles solution ~ 1OD/ ml nanoparticles. After 20 minutes incubation at room temperature, phosphate buffer and sodium dodecyl sulfate, were added to final concentration of 0.01 M and 0.1 % respectively. Then, salting process was done by adding 3 portions of sodium chloride solution at 30 minutes intervals to bring the final concentration of sodium chloride to 0.3 M. The solution was incubated overnight with very gentle shaking. Centrifugation (14,000 rpm for 30 minutes) was performed 5 times for washing and removing the excess probes; the unconjugated probes concentration was measured in the supernatant to calculate their number. The gold pellet was re-suspended in three mL of assay buffer (10 mM phosphate, 150 mM NaCl and 0.1% SDS, pH= 7.4). The color of the solution containing the functionalized AuNPs was not different from the original AuNPs and no aggregation was observed, indicating the success of the conjugation process. In a side reaction, the aforementioned procedures were done exactly for Topoisomerase 1& 2 alfa (TOPO1, & TOPO2 alfa), Tyrosyl- DNA phosphodiesterases 1&2 (TDP1 and TDP2) thiolated probes. Charge and size of the functionalized AuNPs (nanoprobes) was determined by Dynamic light scattering (DLS).

3. Nanoparticles size and charge measurements (Dynamic light scattering measurements)

The size and charge of the prepared AuNPs in addition to some samples (both HCV positive and negative samples) were recorded by the DLS. Each sample was measured 3 times and

scanned 100 times for each measurement, the mean and standard deviation for each sample was calculated by the instrument

Results

a

		Size (d.nm):	% Intensity:	St Dev (d.nm):
Z-Average (d.nm): 20.95	Peak 1:	48.76	87.3	33.95
PDI: 0.577	Peak 2:	4.365	12.7	1.546
Intercept: 0.830	Peak 3:	0.000	0.0	0.000

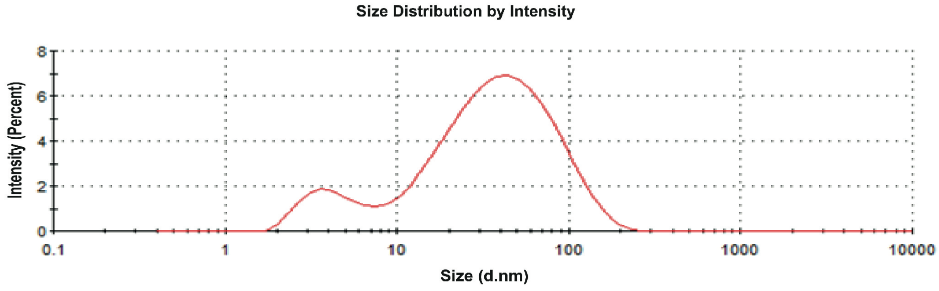

b

		Mean (mV)	Area (%)	St Dev (mV)
Zeta Potential (mV): -48.5	Peak 1:	-47.1	94.3	10.2
Zeta Deviation (mV): 12.8	Peak 2:	-78.1	2.7	3.58
Conductivity (mS/cm): 1.23	Peak 3:	-88.1	2.6	3.06

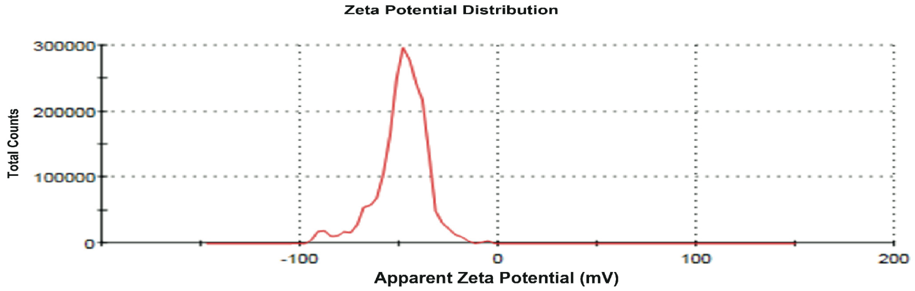

Figure S1: (a) Size of citrate AuNPs. The mean average size is about 20 nm, with standard deviation 0.676 (b) the charge of the citrate AuNPs was -48, with standard deviation 0.873.

a

			Size (d.nm):	% Intensity:	St Dev (d.nm):
Z-Average (d.nm):	38.47	Peak 1:	64.89	98.2	43.04
PdI:	0.437	Peak 2:	3.302	1.8	0.6460
Intercept:	0.839	Peak 3:	0.000	0.0	0.000

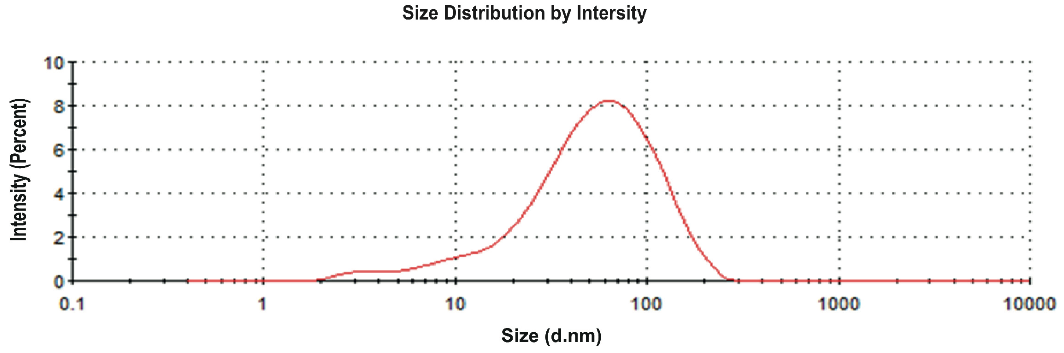

b

			Mean (mV)	Area (%)	St Dev (mV)
Zeta Potential (mV):	-27.7	Peak 1:	-27.0	96.4	10.4
Zeta Deviation (mV):	22.3	Peak 2:	5.00	3.6	3.56
Conductivity (mSLcm):	3.53	Peak 3:	0.00	0.0	0.00

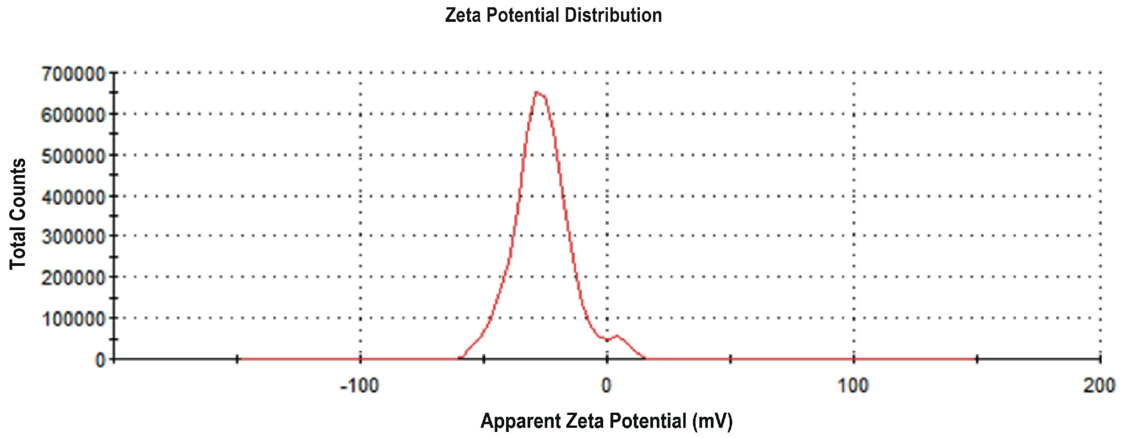

Figure S2: (a) Size of citrate functionalized AuNPs (nanoprobe). The mean average size is about 38 nm, with standard deviation 0.26. Note the increase in the size compared to the citrate AuNPs by about 19 nm after probe functionalization (b) the charge of the nanoprobe is -27 mv, the charge has been decreased from the naked citrate AuNPs, due to probe functionalization, with standard deviation 4.97.

a

			Size (d.nm):	% Internsity:	St Dev (d.nm):
Z-Average (d.nm):	41.86	Peak 1:	50.73	100.0	16.48
PdI:	0.191	Peak 2:	0.000	0.0	0.000
Intercept:	0.904	Peak 3:	0.000	0.0	0.000

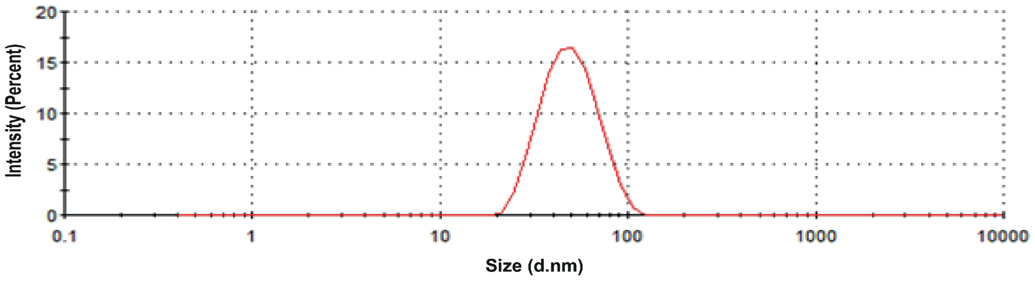

b

				Area (%)	St Dev (mV)
Zeta Potential (mV):	39.2	Peak 1:	39.2	100.0	13.5
Zeta Deviation (mV):	13.5	Peak 2:	0.00	0.0	0.00
Conductivity (mS/cm):	18.0	Peak 3:	0.00	0.0	0.00

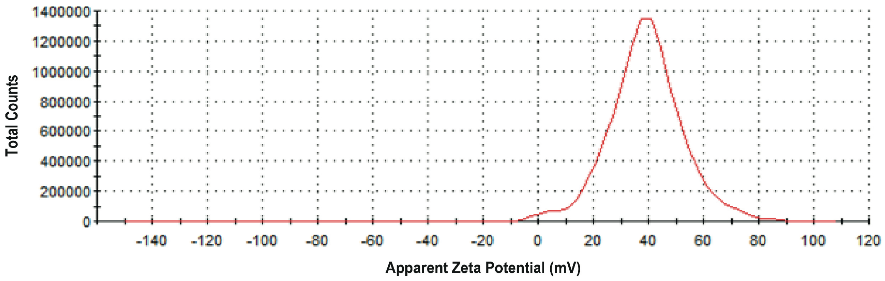

Figure S3: (a) Size of cysteamine AuNPs. The mean average size is about 41 nm, with standard deviation 0.1955 (b) and the charge is +39 mv with standard deviation 0.987.

a

			Size (d.nm):	% Intensity:	St Dev (d.nm):
Z-Average (d.nm):	30.55	Peak 1:	35.69	83.5	19.30
PdI:	0.395	Peak 2:	316.0	12.5	144.2
Intercept:	0.898	Peak 3:	4358	4.0	949.7

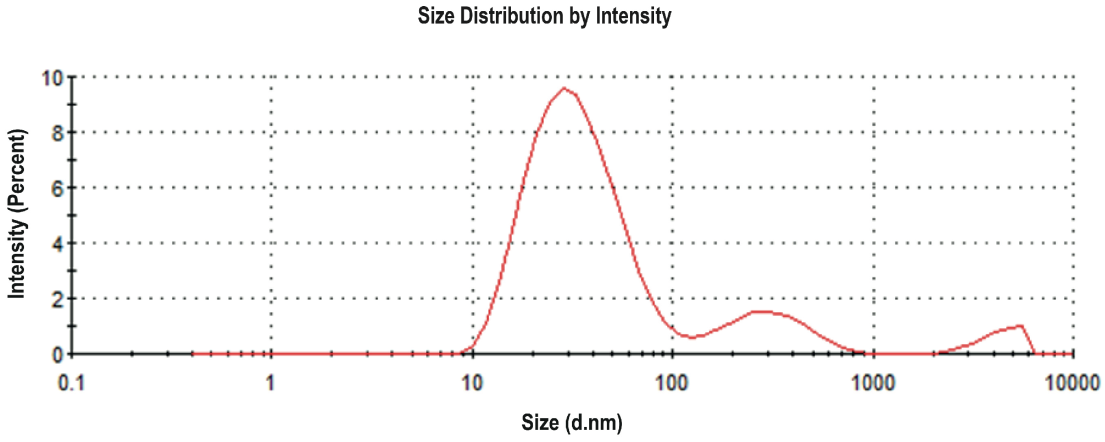

b

			Mean (mV)	Area (%)	St Dev(mV)
Zeta Potential (mV):	99.0	Peak 1:	92.8	79.7	12.3
Zeta Deviation (mV):	17.4	Peak 2:	126	20.3	4.90
Conductivity(mS/cm):	0.209	Peak 3:	0.00	0.0	0.00

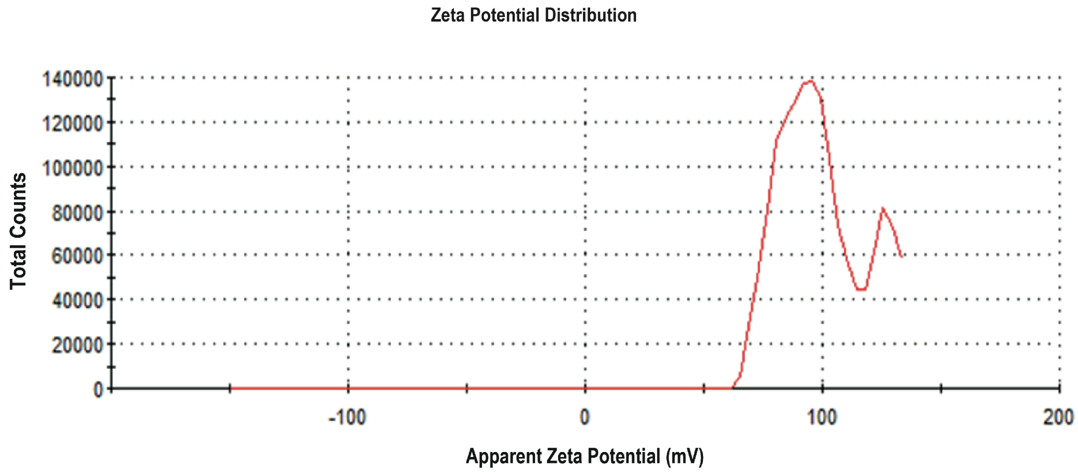

Figure S4: (a) Size of CTAB AuNPs. The mean average size is about 30 nm, with standard deviation 0.767 (b) and its charge is +100 mv with standard deviation 23.4.

a

			Size (d.nm):	% Intensity:	St Dev (d.nm):
Z-Average (d.nm):	189.1	Peak 1:	190.9	93.2	71.81
PdI:	0.294	Peak 2:	4787	6.8	730.9
Inercept:	0.860	Peak 3:	0.000	0.0	0.000

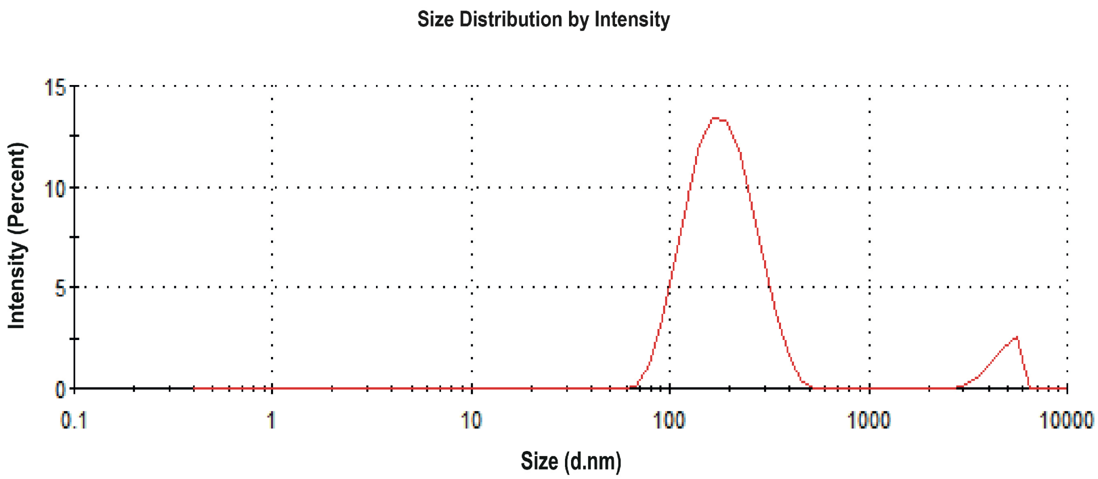

b

			Mean (mV)	Area (%)	St Dev (mV)
Zeta Potential(mV):	-7.52	Peak 1:	-7.52	100.0	4.25
Zeta Deviation (mV):	4.23	Peak 2:	0.00	0.0	0.00
Conductivity (mS/cm):	0.152	Peak 3:	0.00	0.0	0.00

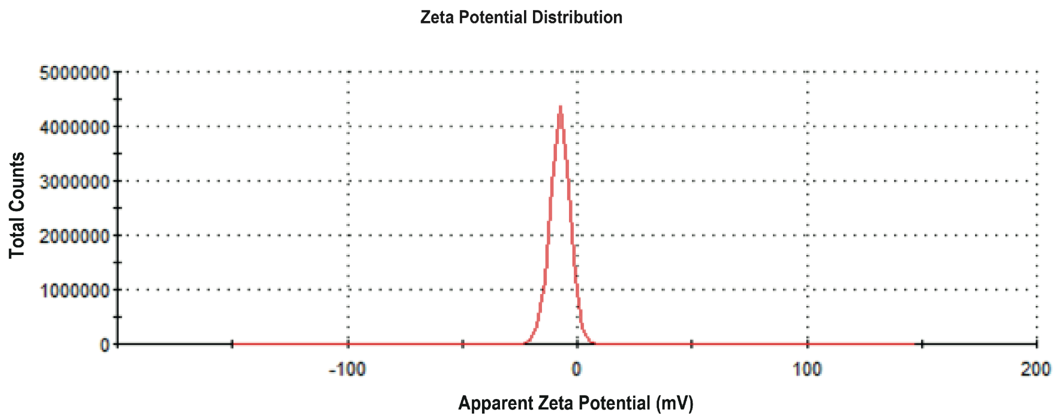

c

			Size (d.nm):	% Intensity:	St Dev (d.nm)
Z-Average (d.nm):	900.1	Peak 1:	324.6	68.8	85.07
PdI:	0.787	Peak 2:	51787	31.2	487.0
Intercept:	0.266	Peak 3:	0.000	0.0	0.000

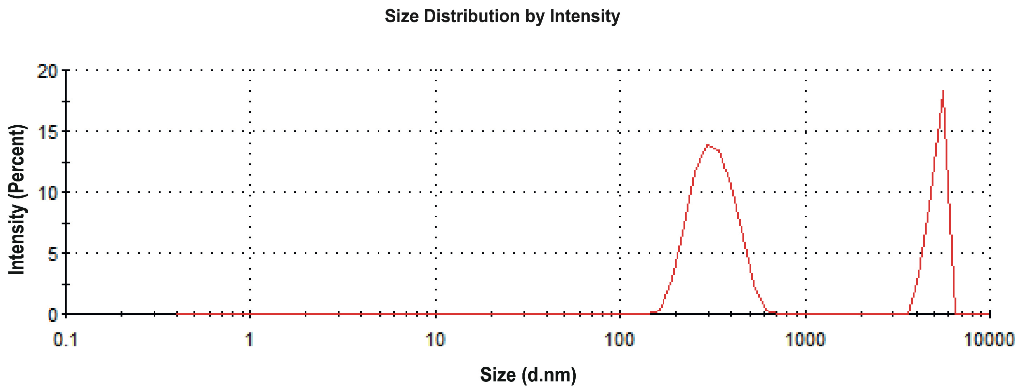

d

			Mean (mV)	Area (%)	St Dev (mV)
Zeta Potential (mV):	12.2	Peak 1:	12.2	100.0	4.69
Zeta Deviantion (mV):	4.69	Peak 2:	0.00	0.0	0.00
Conductivity (mS/cm):	0.122	Peak 3:	0.00	0.0	0.00

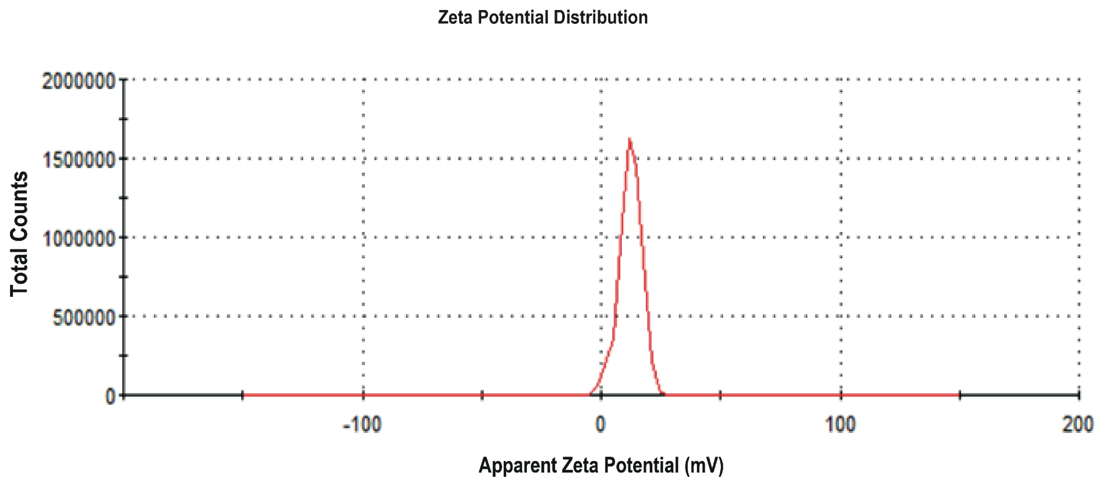

Figure S5: (a) Size of HCV positive sample, after performing the nano-assay directly was calculated to be 198nm and. The increase in positive sample size is attributed to the aggregated population of the AuNPs, which is supported by the positive samples spectrum. (b)The charge is negative (-7.52 mv). The overall negative charge of the sample mixture, confirmed the distribution of the cationic AuNPs onto the folded RNA phosphate backbone. (c) Size of HCV negative sample, after performing the assay directly, which increased significantly to ~900 nm, indicating nanoparticles aggregation due to absence of the HCV RNA. (d) The charge is positive (12.2 mv), indicating cationic AuNPs aggregation onto the nanoprobe, leading to decrease in their interparticle distance, and so, the overall charge was positively charged.

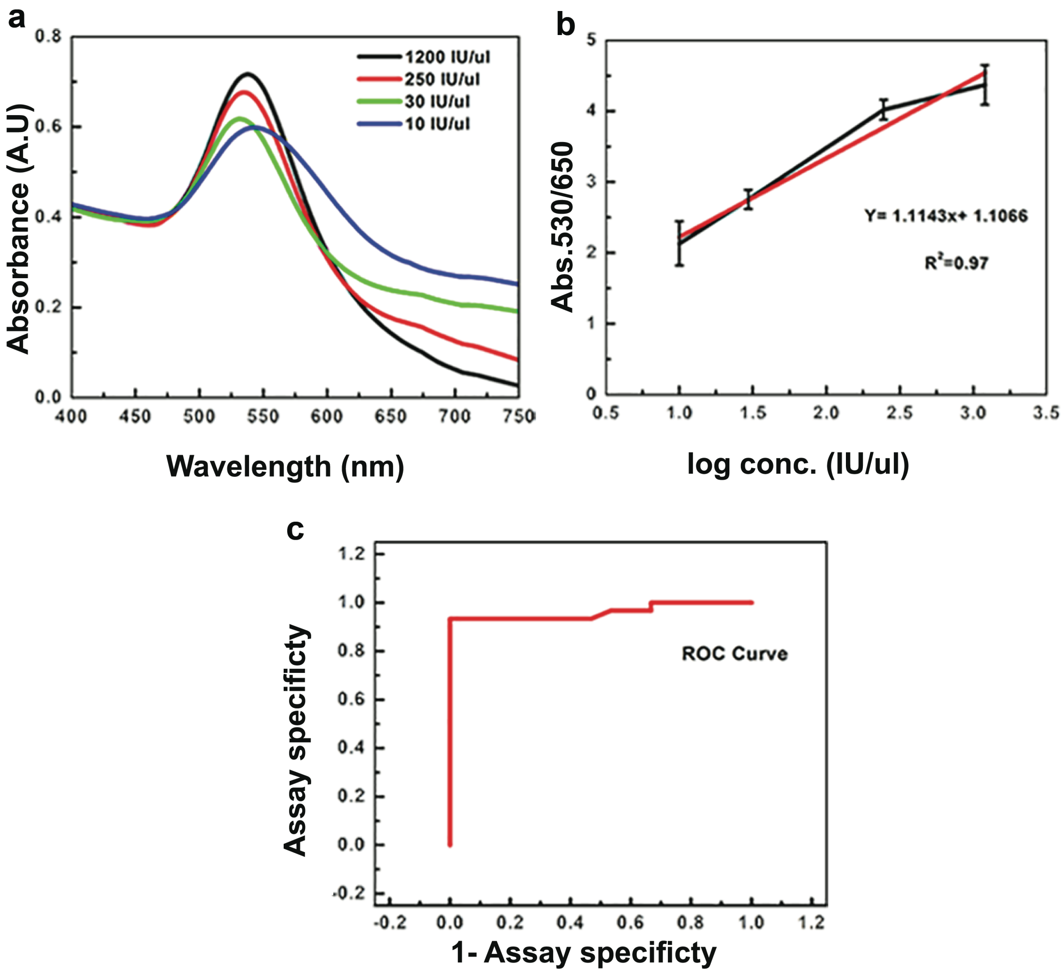

Figure S6: Quantification of HCV RNA in serum clinical samples. (**a**) The extinction spectra of samples with serial dilutions from 10 IU/µl up to 1200 IU/µl. The total reaction volume was 50 µl and 10 µl was taken from the sample for each measurement. Note that the SPR (λ_{530}) decreases by decreasing the concentration, with a concomitant increase and broadness of the absorbance above 600 nm, according to the RNA concentration, as the concentration decreases, the λmax decreases and shifted to longer wavelength, and become broad (**b**) The standard curve plot of the ratio of the absorbance value for the non-aggregated AuNPs at 530 nm to the aggregated AuNPs at 650 nm at the Y-axis against the HCV RNA log concentration at the X-axis. The relation is linear with correlation coefficient R^2= 0.97. The error bars indicate the standard deviation (SD), of two successive measurements, and each point represent the mean ± SD. The equation has been used to calculate the viral load of all HCV samples performed by the developed nano-assay. (**c**) The ROC curve plot performed by the SPSS statistics 20 software. The sensitivity was plotted at the Y-axis against 1- specificity at the X-axis. The area under the curve was 0.961 with a standard error of 0.029.

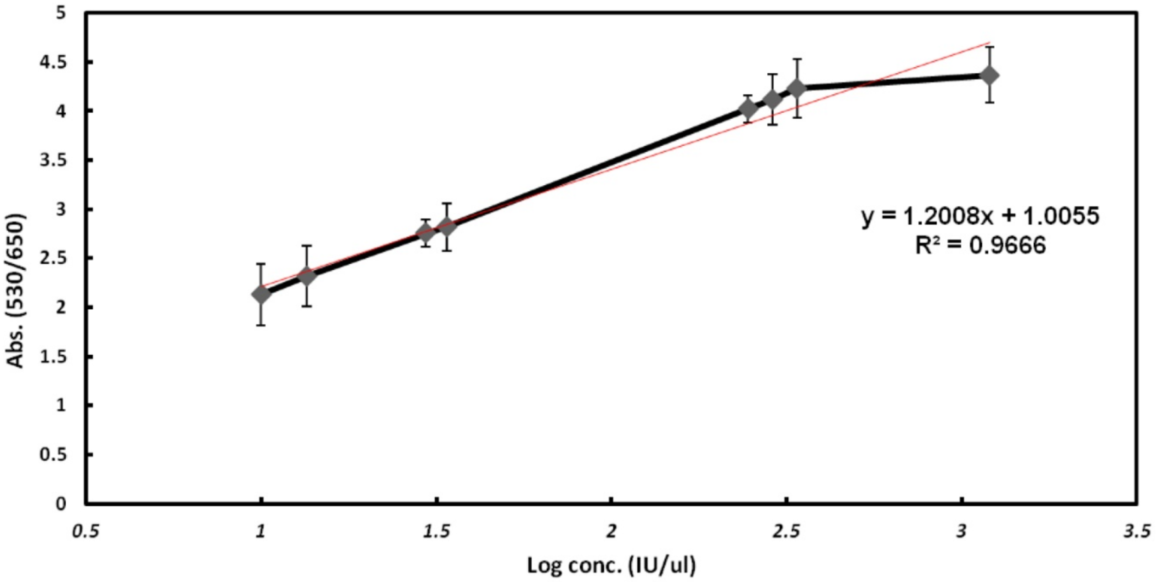

Figure S7: The absorbance ratio (530/650) is plotted as a function of HCV log concentration (IU/µl), for different HCV RNA concentrations. The relation as shown is linear with correlation coefficient R^2= 0.966. The error bars indicate the standard deviation (SD), of two successive measurements, and each point represent the mean ± SD.

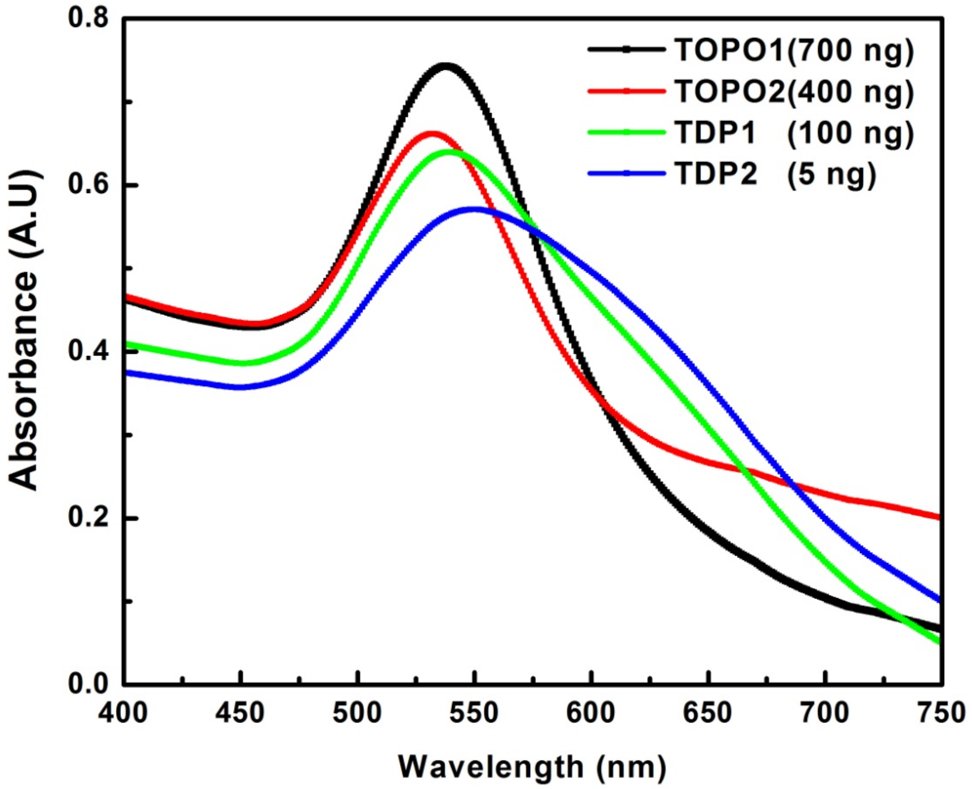

Figure S8: UV-Vis absorption spectra of the AuNPs in the presence of different messenger RNAs (transcripts) extracted from human colon cell lines of different concentrations for each transcript, after performing the nano-assay. The concentrations are expressed in nanogram as determined by their absorbance at 260 nm. As shown, the spectral behavior is quite similar to that of the HCV RNA shown before. The λmax decreases and shifts to longer wavelength as the concentration decrease. These transcripts were extracted using total RNA extraction silica column based kit and then purified by home-made magnetic nanoparticles to increase their purity, and exclude the interference of other RNAs molecules from the cell lines. According to the shown spectral behavior, the developed nano-assay could be used in the detection and quantification of any nucleic acids. Optimization is necessary for performing the assay on nucleic acids from cell lines and/or biopsies to acquire the best possible result. **TOPO1:** Topoisomerase 1, **TOPO2:** Topoisomerase 2alfa, **TDP1:** Tyrosyl- DNA phosphodiesterases 1, and **TDP2:** Tyrosyl- DNA phosphodiesterases 2.

Table S1: comparative table between the nano assay and Real Time-RT-PCR. All the samples performed by the nano assay were compared to Real-Time RT-PCR regarding the viral load. Total RNA from 200 µl of clinical serum samples, was extracted and viral load was determined using HCV TaqMan probe assay, following the manufacturer instructions , through the generation of the Kit standard curve. The quantity expressed in IU/µl was calculated from the standard curve and then expressed as IU/ml. The same samples were done by the developed nano-assay and quantified using the nano-assay standard curve. As shown, samples viral load calculated by the nano-assay is close to the viral load calculated by the Real-Time RT-PCR, and no significant difference between the two methods. The Relative Standard Deviation (RSD) values, of two successive measurements for each sample, using the developed nano-assay were in the range of 1.35-18.24%, and the calculated accuracy was 95.5% indicating that the nano-assay shows good reproducibility, and accuracy respectively. Accuracy was calculated by the addition of the True positive (TP) & True negative (TN) over the total number of samples (n), multiplied by 100 [(TP+TN/n)*100]. (Two HCV positive samples were negative by the nano-assay and considered as false negative, while one of them was negative by the Real Time-RT-PCR and considered as false negative. All the samples were IgG positive. The negative serum samples were all negative when tested with the Real-Time RT-PCR and the nano-assay.

**Samples**	**Anti HCV IgG**	**Viral Load Real-Time RT-PCR**		**Viral Load nano-assay**	
		**IU/µl**	**IU/ml**	**IU/µl**	**IU/ml**
**Sample 1**	positive	85.9	36800	91.7	33047
**Sample 2**	positive	36.7	13787	40.1	14456
**Sample 3**	positive	15.12	5444	14.8	5360
**Sample 4**	positive	29.5	10600	30.6	11050
**Sample 5**	positive	19.4	6986	20.7	7461
**Sample 6**	positive	13.4	4823	13.7	4934

**Sample 7**	Positive	31	13300	34.3	12253
**Sample 8**	Positive	17.5	6560	13.7	4934
**Sample 9**	Positive	No amplification determined		Gold nanoparticles aggregation	
**Sample 10**	Positive	28.7	12300	28.8	10385
**Sample 11**	Positive	25.07	9026	25.4	9174
**Sample 12**	Positive	56.7	20400	Gold nanoparticles aggregation	
**Sample 13**	positive	48.3	17400	43.6	15702
**Sample 14**	positive	25	10700	23.4	8446
**Sample 15**	positive	74.9	27000	61.9	22314
**Sample 16**	positive	45.1	16200	45.4	16365
**Sample 17**	positive	102	43600	101.7	36645
**Sample 18**	positive	40.4	14600	40.1	14456
**Sample 19**	positive	50.6	18200	48.3	17412
**Sample 20**	positive	78.7	33700	84.5	30425
**Sample 21**	positive	35.85	13450	33.3	12002
**Sample 22**	positive	77.9	28000	82.7	29802
**Sample 23**	positive	14	5990	12.8	4638
**Sample 24**	positive	53.2	19100	48.3	17412
**Sample 25**	positive	67.23	25211	68.7	24743
**Sample 25**	positive	40.2	15100	39.3	14161

**Sample 26**	positive	6.93	2500	6.9	2494
**Sample 27**	positive	293.4	125759	292	105155
**Sample 28**	positive	423.0	15229	344.6	124063
**Sample 29**	positive	78.9	28420	81.09	29192
**Sample 30**	positive	32.9	11862	29.4	10602
**Sample 31**	positive	21.2	7632	17.2	6192
**Sample 32**	negative	No amplification determined		Gold nanoparticles aggregation	
**Sample 32**	negative				
**Sample 34**	negative				
**Sample 35**	Negative				
**Sample 36**	Negative				
**Sample 37**	Negative				
**Sample 38**	Negative				
**Sample 39**	Negative				
**Sample 40**	Negative				
**Sample 41**	Negative				
**Sample 42**	Negative				
**Sample 43**	Negative				
**Sample 44**	Negative				
**Sample 45**	Negative				

Table S2: Co-ordinates generated from the SSPS package used in the determination of the detection limit, sensitivity and specificity of the nano-assay. The nano-assay detection limit, sensitivity and specificity row is highlighted in yellow. The detection limit was further confirmed experimentally, through performing the nano-assay on serial dilutions of HCV RNA till 1 IU/µl. The color and the spectral absorbance of the different concentrations were followed. The color started to change to blue (aggregation of the AuNPs) at about 4 IU/µl. This is in consistence with the ROC curve data shown here.

Positive if Greater Than or Equal to	Sensitivity	Specificity
**-.8321**	1.000	1.000
**.2135**	1.000	.867
**.2824**	1.000	.800
**.4039**	1.000	.733
**.5352**	1.000	.667
**.6483**	.967	.600
**.8027**	.967	.533
**.9668**	.967	.467
**1.0676**	.933	.400
**1.2304**	.933	.333
**1.3966**	.933	.267
**1.4409**	.933	.200
**1.6029**	.933	.067
**1.9793**	.933	.000
**4.5769**	.933	.000
**9.9072**	.900	.000
**13.2961**	.867	.000
**14.2990**	.800	.000
**16.0488**	.767	.000
**18.9669**	.733	.000
**22.0943**	.700	.000
**24.4737**	.667	.000
**27.1673**	.633	.000
**29.1499**	.600	.000
**30.0727**	.567	.000
**32.0181**	.533	.000
**33.6891**	.500	.000
**36.6867**	.467	.000
**39.7469**	.433	.000
**41.8883**	.367	.000
**44.5394**	.333	.000

**46.9140**	.300	.000
**55.1759**	.233	.000
**65.3581**	.200	.000
**75.7587**	.167	.000
**83.6494**	.133	.000
**88.1561**	.100	.000
**96.7957**	.067	.000
**196.9453**	.033	.000
**293.0975**	.000	.000

References

Hurst, S.J., Lytton-Jean, A.K., Mirkin, C.A., 2006. Anal Chem 78,8313-8318.

Liu, X., Atwater, M., Wang, J., Huo, Q., 2007. Colloids Surf B Biointerfaces 58,3-7.
